# Seasonal blood chemistry response of sub-tropical nearshore fishes to climate change

**DOI:** 10.1093/conphys/cou028

**Published:** 2014-07-29

**Authors:** Aaron D. Shultz, Zachary C. Zuckerman, Heather A. Stewart, Cory D. Suski

**Affiliations:** 1 Department of Natural Resources and Environmental Sciences, University of Illinois, 1102 S. Goodwin Avenue, MC 047, Urbana, IL 61801, USA; 2 Flats Ecology and Conservation Program, Cape Eleuthera Institute, Eleuthera, The Bahamas

**Keywords:** Blood chemistry, multiple stressors, nearshore, physiological response, stress, temperature

## Abstract

Climate change due to anthropogenic activity will continue to alter the chemistry of the oceans. Future climate scenarios indicate that sub-tropical oceans will become more acidic, and the temperature and salinity will increase relative to current conditions. A large portion of previous work has focused on how future climate scenarios may impact shell-forming organisms and coral reef fish, with little attention given to fish that inhabit nearshore habitats; few studies have examined multiple challenges concurrently. The purpose of this study was to quantify the blood-based physiological response of nearshore fishes to a suite of seawater conditions associated with future climate change. Fish were exposed to an acute (30 min) increase in salinity (50 ppt), acidity (decrease in pH by 0.5 units) or temperature (7–10°C), or temperature and acidity combined, and held in these conditions for 6 h. Their physiological responses were compared across seasons (i.e. summer vs. winter). Bonefish (*Albula vulpes*) exposed to environmental challenges in the summer experienced a suite of blood-based osmotic and ionic disturbances relative to fish held in ambient conditions, with thermal challenges (particularly in the summer) being the most challenging. Conversely, no significant treatment effects were observed for yellowfin mojarra (*Gerres cinereus*) or checkered puffer (*Sphoeroides testudineus*) in either season. Together, results from this study demonstrate that acute climate-induced changes to thermal habitat will be the most challenging for sub-tropical fishes (particularly in the summer) relative to salinity and pH stressors, but significant variation across species exists.

## Abstract (precis)

This experiment uses environmental challenges associated with global climate change to assess the stress response of mangrove fish (bonefish, checkered puffer, and yellowfin mojarra) in two seasons. Identifying species that are most susceptible to environmental stressors will enhance our ability to predict change in ecosystems.

## Introduction

Since the industrial revolution, anthropogenic disturbances, such as the burning of fossil fuels and deforestation, have resulted in an increase in atmospheric carbon dioxide (CO_2_). Carbon dioxide levels have exceeded historical concentrations over the past 650 000 years and have culminated in changes to global climate (Trenberth *et al.*, 2007). In addition to warmer temperatures, climate change also alters the chemistry of the oceans through changes in the evaporation–precipitation cycle (Stott *et al.*, 2008; Feely *et al.*, 2009). Salinity of the ocean is expected to increase in sub-tropical regions as a result of locally reduced precipitation as the planet warms (Gilman *et al.*, 2008). Future climate change predictions indicate that tropical hurricanes will increase in intensity and frequency, resulting in greater amounts of freshwater runoff into nearshore areas during these storms (Knutson *et al.*, 2010). Recent research has also shown that pH decreases by a mean of ∼0.3 units during the rainy season relative to the dry season (Sousa *et al.*, 2013), and this drop in pH is likely to be exacerbated as storms increase in intensity and frequency. Current worst-case scenario predictions by the Intergovernmental Panel on Climate Change (IPCC) indicate that ocean temperatures are expected to increase by 0.3–2.0°C over the next 100 years (Stocker *et al.*, 2013). Taken together, seawater quality parameters (i.e. salinity, pH and temperature) will become more extreme and variable as the climate changes.

Recent research has unequivocally demonstrated tropical marine organisms to be sensitive to future climate change scenarios, with expected negative consequences mainly due to three processes. First, a decrease in pH has been shown to increase mortality in marine zooplankton, disrupt metabolite concentrations in fish and reduce growth rates in invertebrates (Yamada and Ikeda, 1999; Kurihara and Shirayama, 2004; Rodrigues *et al.*, 2013). Second, an increase in temperature has been shown to reduce available dissolved oxygen and concomitantly increase the rate of metabolic processes, which together can reduce the capacity of organisms to perform aerobically (Pörtner and Farrell, 2008; Munday *et al.*, 2009). Third, organisms in the tropics experience a relatively narrow range of temperatures annually compared with organisms in temperate regions and have adapted to these narrow thermal environments to minimize maintenance costs, resulting in organism-specific thermal niches that can overlap (Pörtner and Farrell, 2008; Huey *et al.*, 2009). At present, a large proportion of recent research examining the impact of future climate-induced changes on tropical marine ecosystems has focused on calcifiers (shell-forming organisms), invertebrates, coral and fish that inhabit either open ocean or coral reef ecosystems, with little effort devoted to other marine ecosystems (Przeslawski *et al.*, 2008; Hofmann *et al.*, 2010). More physiological and ecological research on fish from different habitats in the tropics is needed to improve our understanding of and ability to predict how tropical marine ecosystems will respond to future climate change (Roessig *et al.*, 2005).

Nearshore habitats are characterized by dynamic abiotic conditions that fluctuate over short periods of time, including diurnal periods of seawater inundation and drying. Moreover, fluctuations in the levels of a number of abiotic conditions, such as salinity, temperature, pH, oxygen and carbon dioxide content, vary depending on the time of day, tidal cycle and season (Lam *et al.*, 2006). The ability of fish to respond to this dynamic abiotic environment relies on the co-ordination of internal components (e.g. cells, organelles and tissues) and processes (e.g. intra- and extracellular acid–base chemistry) to maintain homeostasis. A disruption in one of these components or processes sets the physiological limits for the whole organism, and identifying which species demonstrate the greatest whole-organism sensitivity to environmental challenges will be important when evaluating limits to climate change (Somero, 2010). Currently, little information exists on the blood chemistry of species of fish that inhabit nearshore ecosystems, much less their physiological response to environmental challenges associated with climate change (Lam *et al.*, 2006).

Based on this background, the purpose of this study was to assess the relative impacts of climate change stressors on several fish species in the nearshore ecosystem (i.e. a community approach) and identify the physiological mechanisms that respond to these stressors. To do this, the blood-based physiological response of bonefish (*Albula vulpes*), checkered puffer (*Sphoeroides testudineus*) and yellowfin mojarra (*Gerres cinereus*) was quantified after exposure to seawater conditions associated with future climate change. Specifically, fish were exposed to an acute increase in salinity, acidity or temperature, or temperature and acidity combined, and their responses were compared across seasons (i.e. summer vs. winter). Results from this study will improve our understanding of how nearshore fish will cope with future climate change, indicate which species are more susceptible to changes in environmental conditions and identify which component of future climate change scenarios will be most challenging for nearshore fishes.

## Materials and methods

### Study site

This study was conducted at a remote field station, The Cape Eleuthera Institute (CEI), in Eleuthera, The Bahamas (24°50′05″ N 76°20′32″ W). All research conformed to the University of Illinois Institutional Animal Care and Use Committee protocol (Protocol # 09160). Adult bonefish (summer fork length, mean ± SEM 382 ± 5 mm, range = 300–452 mm; and winter fork length, mean ± SEM 428 ± 6 mm, range = 362–506 mm), checkered puffer (summer total length, mean ± SEM 191 ± 3 mm, range = 145–240 mm; and winter total length, mean ± SEM 158 ± 11 mm, range = 164–244 mm) and juvenile yellowfin mojarra (summer fork length, mean ± SEM 129 ± 3 mm, range = 105–185 mm; and winter fork length, mean ± SEM 158 ± 4 mm, range = 87–210 mm) were captured by seining local tidal creeks near CEI and transferred to plastic 76 l totes filled with ambient sea water. Fish were transported by boat to the CEI wetlab in <30 min, and seawater in the totes was exchanged every 5 min (Murchie *et al.*, 2009).

Upon arrival at the CEI aquatic facility, fish were transferred to two large holding tanks (3.7 m diameter × 1.25 m height; 13 180 l) continuously supplied with fresh seawater (1800 l h^−1^) and aerated with a low-pressure pump (Sweetwater model S41; 15 V; 3450 rpm; Aquatic Ecosystems, Apopka, FL, USA). Dissolved oxygen (in milligrams per litre), salinity (in parts per thousand, ppt), acidity (pH) and temperature (in degrees Celsius) were monitored regularly during holding (YSI 55, 85, pH10A, Yellow Springs, OH, USA; Table 1). All fish were acclimated to laboratory conditions for a minimum of 48 h prior to experimentation. During holding, fish were fed frozen sardines (*Sardenella aurita*) to satiation, but were starved for 24 h prior to experimentation.

**Table 1:** Mean daily water conditions for laboratory holding tanks and two tidal creeks across seasons

Location	Season	Descriptive statistic	Dissolved oxygen (mg l^−1^)	Salinity (ppt)	Acidity (pH)	Temperature (°C)
Holding tank	Summer	Mean ± SEM	5.64 ± 0.1	38.7 ± 0.7	8.1 ± 0.1	29.4 ± 0.9
		Range	5.06–6.08	38.1–39.7	7.9–8.2	28.0–30.6
		*n*	7	7	7	7
	Winter	Mean ± SEM	5.64 ± 0.1	41.9 ± 0.1	8.1 ± 0.1	20.8 ± 0.6
		Range	4.71–7.27	41.2–42.8	7.9–8.2	16.8–25.6
		*n*	7	7	7	7
Tidal creeks	Summer	Mean ± SEM	5.66 ± 0.1	36.2 ± 0.3	8.3 ± 0.06	30.3 ± 0.06
		Range	2.46–8.71	17.2–40.5	7.3–9.3	23.5–43.0
		*n*	118	119	49	2922
	Winter	Mean ± SEM	3.75 ± 0.2	41.5 ± 0.4	9.2 ± 0.1	21.2 ± 0.08
		Range	2.64–4.64	40.4–43.2	8.9–9.9	11–35.7
		*n*	11	11	11	2850

Holding tank conditions in the summer were measured from 5 to 15 August 2009 and in the winter from 12 February to 13 March 2010. Dissolved oxygen, salinity and acidity in the tidal creeks were measured at the mouth in the summer from 3 June to 14 July 2011 and in the winter from 27 January to 15 February 2011. Temperature was recorded at the mouth in the summer from 1 June to 31 August 2011 and in the winter from 27 January to 27 March 2011.

To quantify ambient water characteristics in nearshore ecosystems, seawater parameters were measured in two tidal creeks in the winter and summer. A single autonomous temperature logger (iButton DS1923; Maxim, Dallas, TX, USA) was deployed in the mouth of each collection creek at a depth of ∼0.5 m (at low tide), and temperature (±0.5°C) was sampled hourly. Dissolved oxygen, salinity and pH were measured several times per week during both the summer and winter sampling periods (Table 1).

### Acute response to climate change stressors

To quantify the response of nearshore fishes to acute changes in environmental conditions, fish were exposed to one of the following four separate challenges: (i) increase in salinity; (ii) decrease in pH; (iii) increase in temperature; and (iv) temperature increase coupled with a concurrent decrease in pH (referred to as T + pH). The environmental challenges exceeded the predictions of future oceanic conditions generated by the IPCC, but were still representative of conditions for nearshore ecosystems (Trenberth *et al.*, 2007; Table 2). To accomplish the environmental challenges, fish were transferred from large holding tanks into individual, aerated plastic totes, scaled according to fish size (bonefish, 76 l; checkered puffer and yellowfin mojarra, 14 l) resting in a raceway (3.09 m length × 0.65 m width × 0.17 m height), and allowed to acclimate for a minimum of 12 h prior to experimentation. The individual totes were continuously supplied with recirculating seawater (Eheim pump 1046A; 5 l min^−1^) from a reservoir tank (Igloo cooler 108 l), completing a closed water system (Vanlandeghem *et al.*, 2010). Treatment levels for the environmental challenges were attained by gradually adjusting seawater conditions over a 30 min period to target conditions, and then maintaining these target conditions for 6 h. An acute change in seawater conditions has been used to assess the sensitivity of fish species to climate change stressors (Gräns *et al.*, 2013).

**Table 2:** Water quality conditions for bonefish, yellowfin mojarra and checkered puffer held for 6 h in altered seawater conditions in the summer and winter

Season	Species	Treatment	Water quality parameter	Mean	SEM
Summer	All species	Control	Dissolved oxygen (mg l^−1^)	5.93	0.07
			Salinity (ppt)	39.7	0.33
			Acidity (pH)	8.02	0.03
			Temperature (°C)	28.2	0.47
		Salinity	(ppt)	49.8	0.19
		Acidity	(pH)	7.57	0.01
	Bonefish	Temperature	(°C)	35	0.11
		Temperature and acidity	(°C)	35	0.06
			(pH)	7.55	0.02
	Checkered puffer	Temperature	(°C)	37.6	0.03
		Temperature and acidity	(°C)	37.7	0.23
			(pH)	7.5	0.09
	Yellowfin Mojarra	Temperature	(°C)	37.6	0.08
		Temperature and acidity	(°C)	38.1	0.11
			(pH)	7.37	0.12
Winter	All species	Control	Dissolved oxygen (mg l^−1^)	6.8	0.09
			Salinity (ppt)	42.6	0.04
			Acidity (pH)	8.71	0.05
			Temperature (°C)	20.7	0.22
		Salinity	(ppt)	49.5	0.62
		Acidity	(pH)	8.0	0.02
	Bonefish	Temperature	(°C)	27.5	0.08
		Temperature and acidity	(°C)	27	0.14
			(pH)	8.02	0.01
	Checkered puffer	Temperature	(°C)	33.2	0.53
		Temperature and acidity	(°C)	33.2	0.45
			(pH)	7.8	0.04
	Yellowfin Mojarra	Temperature	(°C)	32.2	0.14
		Temperature and acidity	(°C)	33.4	0.54
			(pH)	7.97	0.04

Water quality values were pooled across species for control, salinity and acidity treatments.

Salinity was increased from 36 to 50 ppt by dissolving sea salt (Instant Ocean; Aquatic Ecosystems, Apopka, FL, USA) in seawater and then mixing it into the reservoir tank (Haney and Walsh, 2003); salinity was verified using a hand-held meter (YSI 85).

The pH was decreased from 8.1 ± 0.06 (mean ± SEM; range = 8.2–7.9) to 7.54 ± 0.05 (mean ± SEM; range = 7.7–7.4) by transferring small amounts (1–3 ml) of 31.45% HCl (muriatic acid; Sunnyside Corporation, Wheeling, IL, USA) into the reservoir tank, in a similar manner to Kurihara and Shirayama (2004). These conditions were maintained by transferring HCl into the reservoir tank as needed (HCl was used to decrease the pH of the water instead of CO_2_ because cylinders of compressed CO_2_ were not available at this remote field station).

Temperature was increased by using immersion heaters in the main reservoir and distributing warmed water to fish in the plastic totes (Vanlandeghem *et al.*, 2010). Previous work has shown that upper lethal temperature, incipient lethal temperatures and chronic thermal stress for animals can vary seasonally, partly as a result of acclimation/acclimatization (Murchie *et al.*, 2011), which tracks with seasonal increases/decreases in oceanic temperatures. For the present experiment, an absolute thermal maximum treatment for each species was not used; rather, temperature levels for the thermal treatment were 7°C above ambient conditions for bonefish and 10°C above ambient conditions for checkered puffers and yellowfin mojarra. These values go beyond the predictions for sea surface temperatures of oceans projected by the IPCC (Stocker *et al.*, 2013), but are not unrealistic temperature values for nearshore ecosystems (Table 1). The same change in temperature was used for this treatment in the winter (e.g. ambient seawater at 20°C was increased to 27°C for bonefish).

For the T + pH treatment, temperature was simultaneously increased by the addition of immersion heaters, while the pH was decreased by the addition of HCl to the reservoir (bonefish, 7°C and 0.5 pH units; checkered puffers and yellowfin mojarra, 10°C and 0.5 pH units).

Fish in the control treatment were handled in an identical manner to the experimental fish described above, except that water conditions were not altered.

### Blood sampling and analysis

Briefly, blood samples were drawn from fish using a heparinized 22 gauge needle attached to a 1 ml syringe inserted into the caudal vessel, following the 6 h exposure to an environmental challenge or control conditions. The samples were transported to a laboratory at the University of Illinois, and the following blood parameters were quantified: haematocrit, potassium (K^+^), sodium (Na^+^), chloride (Cl^−^), calcium (Ca^2+^), glucose and cortisol (for details see Shultz *et al.* 2011). These blood parameters have been shown to change in marine fish exposed to temperature, pH and salinity challenges (Ishimatsu *et al.*, 2004; Evans *et al.*, 2009; Nordlie, 2009).

### Data analysis

Statistical analyses were performed separately for each fish species, with a focus on intraspecific differences in blood chemistry values between seasons. Blood-based metrics were normally distributed and compared using a two-way analysis of variance (ANOVA) with treatment and season as main effects, and treatment × season as an interaction term. Tukey's *post hoc* test was performed when at least one main effect or the interaction term was deemed significantly different. Data analysis was completed using JMP 7.0.2 (SAS Institute, Cary, NC, USA) with α = 0.05.

## Results

### Bonefish

Bonefish exposed to environmental challenges associated with climate change experienced a suite of blood-based physiological disturbances. When compared with control values, plasma Cl^−^ concentrations increased by ∼20% after bonefish were exposed to a 14 ppt increase in salinity (Tables 3 and 4). Likewise, both an increase in temperature by 7°C and an increase in temperature by 7°C coupled with a decrease in pH by 0.5 units resulted in nearly a 25% increase in plasma Cl^−^ concentrations relative to control values. Acidified seawater (decrease of 0.5 pH units) resulted in the greatest increase (33%) in Cl^−^ values when compared with control values. Plasma lactate values increased over 4-fold when fish were held in acidified seawater relative to fish held in ambient conditions (Table 3). Bonefish exposed to an increase in temperature experienced a doubling of plasma glucose concentrations relative to control concentrations (Table 3). None of the treatments in the summer caused Na^+^, K^+^, haematocrit or cortisol values to differ significantly from control values. In contrast, plasma Ca^2+^ was the only variable that changed in the winter, and it decreased by nearly 40% when fish were held in water 7°C warmer than ambient (Table 3).

**Table 3:** Concentrations of different plasma constituents for bonefish following a suite of environmental challenges applied in the summer and the winter

Plasma variable	Season and treatment^†^									
	Summer					Winter				
	C	S	A	T	T + pH	C	S	A	T	T + pH
Na^+^ (mmol l^−1^)	174.3 ± 3.5 (11)	174.7 ± 4.6 (6)	174.8 ± 3.7 (8)	184.0 ± 5.5 (7)	165.1 ± 8.7 (7)	193.9 ± 8.6 (8)	210.7 ± 8.1 (8)	211.6 ± 9.3 (8)	231.9 ± 13.4 (7)	220.4 ± 9.1 (8)
K^+^ (mmol l^−1^)	5.54 ± 0.72 (11)	4.63 ± 0.54 (6)	6.1 ± 0.65 (8)	5.24 ± 0.19 (7)	5.55 ± 0.61 (7)	4.7 ± 0.46 (8)	4.22 ± 0.36 (7)	3.99 ± 0.52 (8)	6.13 ± 0.25 (7)	4.87 ± 0.36 (8)
Cl^−^ (mmol l^−1^)	**154.8**^**a**^ ± **3.6 (11)**	**183.9**^**b**^ ±** 4.1 (7)**	**206.3**^b^* ±** 8.4 (8)**	**195.4**^**b**^ ±** 8.9 (7)**	**191.1**^b^* ±** 3.3 (8)**	158.8 ± 1.7 (8)	179.1 ± 6.9 (8)	172.3 ± 9.8 (8)	168.9 ± 5.1 (8)	160.6 ± 2.5 (8)
Ca^2+^ (mmol l^−1^)	10.8 ± 0.56 (10)	12.3 ± 0.35 (6)	10.6 ± 0.8 (8)	12.4* ±** 0.67 (4)**	11.8 ± 0.11 (6)	**10.8**^**a**^ ± **0.37 (8)**	**10.4**^**a**^ ± **0.39 (8)**	**10.9**^**a**^** **±** 0.75 (8)**	**7.8**^**b**^ ± **0.85 (7)**	**9.8**^**a**^ ± **0.45 (8)**
Cortisol (ng ml^−1^)	25.1 ± 4.7 (11)	35.8 ± 5.3 (7)	37.0 ± 11.1 (8)	50.3 ± 4.7 (4)	35.4 ± 5.7 (7)	9.3 ± 0.9 (8)	17.8 ± 3.0 (8)	15.5 ± 2.2 (8)	10.7 ± 1.8 (7)	13.1 ± 2.9 (7)
Lactate (mmol l^−1^)	**0.29**^**a**^ ± **0.08 (9)**	**0.40**^**a**^ ± **0.13 (6)**	**1.5**^b^* ± **0.3 (8)**	**0.35**^**a**^ ± **0.03 (3)**	**0.97**^**a**^ ± **0.24 (6)**	**0.21**^**a**^ ± **0.07 (8)**	**0.22**^**a**^ ± **0.11 (8)**	**0.07**^**a**^** **±** 0.05 (8)**	**1.4**^**b**^ ± **0.29 (7)**	**0.60**^**a**^ ± **0.18 (8)**
Glucose (mmol l^−1^)	**3.8**^**a**^ ± **0.39 (11)**	**4.0**^**a**^ ± **0.21 (7)**	**3.1**^**a**^ ± **0.39 (8)**	**7.6**^b^* ± **0.68 (8)**	**5.5**^**a**^ ± **0.76 (8)**	3.4 ± 0.17 (8)	3.2 ± 0.40 (8)	3.4 ± 0.30 (8)	4.5 ± 0.20 (7)	4.40 ± 0.37 (8)
Haematocrit (packed cell volume)	0.25 ± 0.01 (11)	0.31 ± 0.04 (7)	0.36 ± 0.05 (5)	0.38 ± 0.02 (8)	0.33 ± 0.02 (7)	0.22 ± 0.01 (8)	0.28 ± 0.02 (8)	0.24 ± 0.01 (8)	0.27 ± 0.01 (6)	0.29 ± 0.03 (8)

The different challenges applied were control (C), an increase in salinity (S), a decrease in pH (A), an increase in temperature (T) or a combination of increased temperature and reduced pH (T + pH). Bonefish were held in these treatments for 6 h prior to blood sampling. Tukey's *post hoc* test was used to compare blood-based stress metrics (sample sizes in parentheses) for bonefish exposed to climate change stressors in the summer and winter. Means with different superscript letters indicate a significant difference between a treatment and the control group within a season. An asterisk denotes a significant difference across seasons for the same treatment. ANOVA results are shown in Table 4.

**Table 4:** Results of a two-way ANOVA, with treatment, season and the treatment × season interaction as effects, comparing the physiological response of bonefish to five treatments in two seasons

Plasma concentrations	Treatment			Season			Treatment × season		
	*F*	d.f.	*P* value	*F*	d.f.	*P* value	*F*	d.f.	*P* value
Na^+^	2.43	4	0.06	**61**.**96**	**1**	**<0**.**0001**	1.61	4	0.18
K^+^	1.24	4	0.30	3.45	1	0.07	1.99	4	0.11
Cl^−^	**9**.**47**	**4**	**<0**.**0001**	**23**.**54**	**1**	**<0**.**0001**	**4**.**26**	**4**	**0**.**0038**
Ca^2+^	0.98	4	0.43	**17**.**93**	**1**	**<0**.**0001**	**4**.**53**	**4**	**0**.**0028**
Cortisol	1.79	4	0.14	**46**.**92**	**1**	**<0**.**0001**	1.23	4	0.30
Lactate	**4**.**99**	**4**	**0**.**0015**	2.6	1	0.11	**9**.**63**	**4**	**<0**.**0001**
Glucose	**14**.**10**	**4**	**<0**.**0001**	**13**.**72**	**1**	**0**.**0004**	**4**.**0**	**4**	**0**.**0056**
Haematocrit	**5**.**17**	**4**	**0**.**0011**	**22**.**15**	**1**	**<0**.**0001**	2.20	4	0.08

Data tested by ANOVA are presented in Table 3.

Several physiological disturbances were observed when climate change stressors were compared across seasons. In the summer, bonefish in the acidity and T + pH treatments displayed an increase of nearly 20% in plasma Cl^−^ concentrations relative to values for fish in the same treatments during the winter (Table 3). Likewise, bonefish exposed to acidified seawater during the summer experienced a 20-fold increase in plasma lactate concentrations relative to fish in the winter. Moreover, bonefish exposed to an increase in temperature during the summer exhibited an increase in plasma glucose and Ca^2+^ levels by ∼60% relative to fish in the same treatment during the winter (Table 3).

### Checkered puffers

No significant interactions between treatment and season were observed when checkered puffers were exposed to climate change stressors in the summer and winter, with significant treatment effects limited to that of season or treatment independently (Tables 5 and 6). Independent of season, haematocrit levels were significantly greater when fish were exposed to an increase in acidity, temperature or T + pH relative to ambient conditions. Glucose and cortisol concentrations were also elevated in fish exposed to an increase in temperature by 10°C when compared with fish held in ambient seawater (Table 6). Independent of treatment, plasma Cl^−^, Na^+^ and K^+^ concentrations were significantly greater in the winter than in the summer. Conversely, haematocrit levels and plasma cortisol concentrations were elevated in the summer relative to the winter (Table 6).

**Table 5:** Concentrations of different plasma constituents for checkered puffer following a suite of environmental challenges applied in the summer and the winter

Plasma variable	Season and treatment^†^									
	Summer					Winter				
	C	S	A	T	T + pH	C	S	A	T	T + pH
Na^+^ (mmol l^−1^)	165.6 ± 5.32 (8)	172.4 ± 2.73 (6)	166.0 ± 2.92 (6)	180.8 ± 2.98 (6)	181.1 ± 2.23 (4)	190.8 ± 12.99 (7)	191.2 ± 9.70 (8)	192.0 ± 10.3 (8)	195.0 ± 16.0 (6)	178.1 ± 10.4 (6)
K^+^ (mmol l^−1^)	4.15 ± 0.24 (8)	4.02 ± 0.46 (6)	3.32 ± 0.20 (6)	4.51 ± 0.47 (6)	4.56 ± 0.32 (4)	5.48 ± 0.55 (7)	5.09 ± 0.39 (8)	4.50 ± 0.32 (8)	5.3 ± 0.58 (6)	5.28 ± 0.79 (5)
Cl^−^ (mmol l^−1^)	138.4 ± 5.97 (8)	141.9 ± 3.24 (6)	142.6 ± 4.8 (7)	151.8 ± 6.52 (6)	156.1 ± 6.67 (4)	159.8 ± 6.04 (6)	178.6 ± 11.8 (7)	159.4 ± 2.51 (7)	167.3 ± 8.17 (6)	168.8 ± 8.91 (6)
Ca^2+^ (mmol l^−1^)	10.13 ± 0.58 (8)	9.82 ± 0.99 (7)	10.5 ± 1.79 (6)	10.2 ± 1.15 (4)	12.5 ± 0.70 (3)	11.4 ± 0.66 (6)	10.3 ± 0.70 (7)	10.3 ± 0.31 (7)	11.3 ± 0.26 (6)	10.8 ± 0.56 (6)
Cortisol (ng ml^−1^)	19.9 ± 3.49 (6)	5.03 ± 1.7 (5)	12.4 ± 2.52 (6)	59.3 ± 21.1 (3)	27.3 ± 9.80 (4)	3.25 ± 0.86 (5)	4.82 ± 2.17 (3)	2.44 ± 0.85 (6)	22.1 ± 6.90 (6)	12.0 ± 2.14 (5)
Lactate (mmol l^−1^)	0.19 ± 0.19 (7)	0.0 ± 0.0 (4)	0.0 ± 0.0 (6)	0.02 ± 0.01 (4)	0.02 ± 0.02 (3)	0.08 ± 0.05 (6)	0.09 ± 0.02 (8)	0.07 ± 0.04 (8)	0.19 ± 0.04 (6)	0.07 ± 0.04 (6)
Glucose (mmol l^−1^)	1.37 ± 0.19 (8)	1.93 ± 0.46 (7)	1.59 ± 0.18 (5)	2.91 ± 0.43 (7)	2.27 ± 0.21 (6)	1.48 ± 0.15 (7)	1.59 ± 0.26 (7)	1.30 ± 0.18 (5)	2.2 ± 0.47 (6)	1.69 ± 0.16 (6)
Haematocrit (packed cell volume)	0.22 ± 0.01 (8)	0.23 ± 0.01 (8)	0.28 ± 0.05 (6)	0.31 ± 0.02 (8)	0.27 ± 0.02 (6)	0.13 ± 0.02 (7)	0.17 ± 0.01 (7)	0.20 ± 0.02 (8)	0.22 ± 0.03 (7)	0.28 ± 0.02 (6)

The different challenges applied were control (C), an increase in salinity (S), a decrease in pH (A), an increase in temperature (T) or a combination of increased temperature and reduced pH (T + pH). Checkered puffers were held in these treatments for 6 h prior to blood sampling. No significant differences were observed between treatments and the control group within a season. Likewise, no significant differences were observed across seasons for the same treatment. Sample sizes are indicated in parentheses. ANOVA results are shown in Table 6.

**Table 6:** Results of a two-way ANOVA, with treatment, season and the treatment × season interaction as effects, comparing the physiological response of checkered puffers to five treatments in two seasons

Plasma concentrations	Treatment			Season			Treatment × season		
	*F*	d.f.	*P* value	*F*	d.f.	*P* value	*F*	d.f.	*P* value
Na^+^	0.34	4	0.85	**7**.**16**	**1**	**0**.**0098**	0.66	4	0.62
K^+^	2.01	4	0.11	**13**.**79**	**1**	**0**.**0005**	0.17	4	0.95
Cl^−^	1.42	4	0.24	**20**.**52**	**1**	**<0**.**0001**	0.88	4	0.48
Ca^2+^	1.23	4	0.31	0.15	1	0.70	1.08	4	0.37
Cortisol	**11**.**38**	**4**	**<0**.**0001**	**18**.**68**	**1**	**<0**.**0001**	2.39	4	0.07
Lactate	0.67	4	0.62	1.03	1	0.32	0.78	4	0.54
Glucose	**4**.**62**	**4**	**0**.**0028**	3.39	1	0.07	0.57	4	0.69
Haematocrit	**8**.**98**	**4**	**<0**.**0001**	**23**.**53**	**1**	**<0**.**0001**	1.55	4	0.20

Data tested by ANOVA are presented in Table 5.

### Yellowfin mojarra

When qualitatively compared with the other two nearshore species examined in this study, yellowfin mojarra experienced fewer physiological disturbances following the 6 h treatments. No significant interactions between season and treatment were observed when yellowfin mojarra were exposed to climate change stressors in the summer and winter. Independent of season, the temperature and T + pH treatment resulted in a significant increase in haematocrit levels relative to control levels (Tables 7 and 8). Independent of treatment, plasma Ca^2+^ and glucose concentrations in the winter were elevated relative to the summer. Conversely, plasma K^+^ levels in the summer were significantly greater than values in the winter (Table 7).

**Table 7:** Concentrations of different plasma constituents for yellowfin mojarra following a suite of environmental challenges applied in the summer and the winter

Plasma variables	Season and treatment^†^									
	Summer					Winter				
	C	S	A	T	T + pH	C	S	A	T	T + pH
Na^+^ (mmol l^−1^)	172.4 ± 2.27 (8)	178.9 ± 5.74 (5)	182.5 ± 5.85 (7)	180.6 ± 2.27 (7)	191.2 ± 3.30 (8)	186.0 ± 9.67 (11)	181.2 ± 5.75 (6)	189.2 ± 5.94 (10)	175.3 ± 12.9 (6)	181.0 ± 4.17 (7)
K^+^ (mmol l^−1^)	5.83 ± 0.35 (8)	5.51 ± 0.45 (5)	4.25 ± 0.32 (7)	6.32 ±0.83 (7)	5.31 ± 0.20 (8)	3.19 ± 0.68 (10)	3.59 ±0.68 (6)	3.90 ± 0.57 (10)	4.24 ± 0.33 (6)	4.36 ± 0.45 (7)
Cl^−^ (mmol l^−1^)	144.8 ± 2.63 (8)	171.2 ± 8.14 (5)	162.6 ± 10.1 (7)	156.5 ± 6.64 (7)	150.7 ± 2.59 (8)	168.5 ± 9.07 (11)	153.4 ± 6.23 (6)	165.3 ± 7.12 (10)	163.2 ± 8.32 (6)	171.4 ± 3.87 (7)
Ca^2+^ (mmol l^−1^)	9.0 ± 0.96 (5)	8.97 ± 0.74 (6)	9.59 ± 0.56 (8)	9.65 ± 0.31 (6)	11.4 ± 0.66 (7)	13.22 ± 1.50 (11)	11.48 ± 0.71 (6)	11.88 ± 0.61 (10)	11.9 ± 0.91 (6)	12.1 ± 0.92 (7)
Cortisol (ng ml^−1^)	43.4 ± 31.8 (7)	26.4 ± 6.47 (7)	29.3 ± 18.1 (6)	417.6 ± 56.8 (6)	21.0 ± 11.8 (8)	62.75 ± 21.4 (11)	33.6 ± 13.3 (6)	27.79 ± 11.3 (10)	53.83 ± 17.94 (6)	37.4 ± 8.18 (7)
Lactate (mmol l^−1^)	0.31 ± 0.12 (7)	0.06 ± 0.04 (5)	0.48 ± 0.41 (7)	0.36 ± 0.12 (8)	0.22 ± 0.13 (7)	0.04 ± 0.03 (11)	0.09 ± 0.06 (6)	0.20 ± 0.11 (10)	0.23 ± 0.11 (6)	0.26 ± 0.08 (7)
Glucose (mmol l^−1^)	3.35 ± 0.21 (8)	2.88 ± 0.39 (7)	3.56 ± 0.23 (8)	3.68 ±0.25 (8)	3.36 ± 0.21 (8)	4.05 ± 0.60 (11)	3.77 ± 0.63 (6)	3.68 ± 0.51 (9)	4.54 ± 0.42 (6)	4.5 ± 0.26 (7)
Haematocrit (packed cell volume)	0.21 ± 0.02 (8)	0.21 ± 0.02 (6)	0.20 ± 0.02 (8)	0.28 ± 0.01 (7)	0.29 ± 0.01 (8)	0.24 ± 0.02 (11)	0.24 ± 0.008 (7)	0.23 ± 0.02 (10)	0.25 ± 0.02 (6)	0.29 ± 0.02 (7)

The different challenges applied were control (C), an increase in salinity (S), a decrease in pH (A), an increase in temperature (T) or a combination of increased temperature and reduced pH (T + pH). Yellowfin mojarra were held in these treatments for 6 h prior to blood sampling. No significant differences were observed between treatments and the control group within a season. Likewise, no significant differences were observed across seasons for the same treatment. Sample sizes are indicated in parentheses. ANOVA results are shown in Table 8.

**Table 8:** Results of a two-way ANOVA, with treatment, season and the treatment × season interaction as effects, comparing the physiological response of yellowfin mojarra to five treatments in two seasons

Plasma concentrations	Treatment			Season			Treatment × season		
	*F*	d.f.	*P* value	*F*	d.f.	*P* value	*F*	d.f.	*P* value
Na^+^	0.62	4	0.65	0.10	1	0.75	1.0	4	0.41
K^+^	1.31	4	0.28	**20**.**21**	**1**	**<0**.**0001**	1.31	4	0.28
Cl^−^	0.33	4	0.86	2.33	1	0.13	2.30	4	0.07
Ca^2+^	0.58	4	0.68	**14**.**08**	**1**	**0**.**0004**	0.77	4	0.55
Cortisol	0.78	4	0.54	1.55	1	0.22	0.21	4	0.93
Lactate	0.84	4	0.50	1.53	1	0.22	0.50	4	0.74
Glucose	0.89	4	0.47	**7**.**49**	**1**	**0**.**0079**	0.43	4	0.78
Haematocrit	**6**.**58**	**4**	**0**.**0002**	1.07	1	0.31	1.02	4	0.40

Data tested by ANOVA are presented in Table 7.

## Discussion

Of the three species of nearshore fish examined, bonefish displayed the greatest degree of physiological disturbances following exposure to the common environmental challenges, with disturbances in the summer being greater than those in the winter. More specifically, plasma Cl^−^ concentrations increased when bonefish were exposed to acidified seawater, salinity and thermal challenges that exceeded the predictions of the IPCC. In addition, bonefish exposed to acidified seawater also experienced an increase in plasma lactate concentrations. An increase in temperature resulted in greater concentrations of glucose in the blood of bonefish, probably to fuel metabolic demands (Wendelaar Bonga, 1997). Overall, salinity, acid, temperature or T + pH applied in the summer resulted in a plasma Cl^−^ imbalance that required bonefish to expend energy to return to homeostasis. Moreover, the acid and temperature treatments produced additional imbalances in metabolites, suggesting that these two challenges are most physiologically difficult for bonefish to cope with during the summer.

Independent of season, checkered puffers exhibited several physiological disturbances when exposed to environmental challenges that exceeded future IPCC ocean scenarios. More specifically, haematocrit increased when fish were exposed to an increase in temperature, acidity or T + pH. An increase in temperature results in an increase in metabolic rate, which means that more oxygen must be delivered to cells to maintain aerobic metabolism (Pörtner, 2012). Checkered puffers increased either the number or the size of their red cells in an effort to bind more oxygen. Independent of treatment, plasma ion concentrations were higher in the winter relative to the summer, while haematocrit and cortisol levels were also higher in the summer than the winter. While the exact mechanism for these seasonal differences is not known, it could be related to annual cycles independent of temperature (Evans, 1984) or to differences in the activity rates or number of pumps in the cell membrane related to temperature and salinity (Fiess *et al.*, 2007; Sardella *et al.*, 2008), and should be the subject of future study.

Independent of season, yellowfin mojarra demonstrated the lowest degree of physiological disturbance when exposed to environmental stressors relative to bonefish and puffer. An increase in haematocrit values was observed when these fish were exposed to an increase in temperature or T + pH. Independent of treatment, Ca^2+^ and glucose concentrations were higher in the winter, and K^+^ concentrations were higher in the summer. Glucose concentrations were higher in the winter relative to the summer.

Previous climate change work has used treatments that represented the worst-case scenario predicted by the IPCC and found considerable physiological disturbances in shell-forming organisms and reef fish. For example, intertidal gastropods exposed to pH and temperature values expected for 2100 (i.e. decrease in pH by 0.3 units and increase in temperature by 5°C) experienced lower shell growth rates and a disruption in metabolic processes (Melatunan *et al.*, 2013). Likewise, cardinal fish (*Ostorhinchus doederleini* and *Ostorhinchus cyanosoma*) and lemon damselfish (*Pomacentrus moluccensis*) found in relatively stable environmental conditions on reefs demonstrated a reduction in metabolic scope when exposed to future climate change scenarios, which can have negative implications for feeding, growth and reproduction (Munday *et al.*, 2009; Nilsson *et al.*, 2010). In contrast to reef environments, abiotic factors (pH, temperature and salinity) in nearshore ecosystems tend to fluctuate daily, seasonally and with precipitation events (Lam *et al.*, 2006; Rummer *et al.*, 2014). For example, seasonal variation in temperatures measured in tidal creeks near CEI ranged from 40°C in the summer to 11°C in the winter. Adult bonefish migrate between the dynamic abiotic environment in the nearshore ecosystem to deeper (>2 m), relatively stable waters, such as coral reef habitats, with the flooding and ebbing of the tides (Murchie *et al.*, 2013), probably avoiding extreme conditions in nearshore ecosystems, which may explain why these fish were relatively less tolerant to climate change stressors. Conversely, checkered puffers and yellowfin mojarra reside almost exclusively in nearshore ecosystems (Layman and Silliman, 2002) and are therefore regularly subjected to a wide range of temperatures and pH levels, making them more tolerant to conditions that exceed future climate change scenarios. This differential response to climate change has the potential to alter fish assemblages in the future by excluding intolerant species from nearshore ecosystems (e.g. bonefish) and/or reducing their population size, while tolerant species (e.g. yellowfin mojarra and checkered puffers) may become more dominant in these systems.

Interestingly, none of the species from the present study experienced an additive or synergistic physiological response when exposed to two climate change stressors in the T + pH treatment. Previous work has documented an additional reduction in aerobic scope when coral reef fish were exposed to an increase in temperature coupled with acidified seawater (i.e. a synergistic effect) compared with the aerobic scope of these fish in ambient seawater and elevated temperatures (Munday *et al.*, 2009). Future research on nearshore fish should focus on the mechanisms that allow them to cope with multiple stressors.

Results from this study indicate that temperature was the most challenging acute stressor associated with future climate change relative to pH, salinity and temperature + pH. Moreover, changes in the summer caused elevated physiological disturbances relative to changes in the winter. Nearshore fish are likely to have a relatively robust ability to regulate osmotic/ionic balances, including pH (Lam *et al.*, 2006). In contrast, elevated temperatures may cause these mechanisms to break down and can cause proteins to denature, which results in physiological disturbances. These problems are most severe in the summer as fishes may be approaching their ‘pejus’ temperatures (Pörtner and Farrell, 2008), thereby reducing their thermal scope. While these changes may not directly result in mortality for nearshore fishes, there may be other sub-lethal consequences, such as altered habitat selection, which may result in increased likelihood of predation or reduced feeding. Additionally, as reviewed by Boeuf and Payan (2001), osmotic regulation accounts for 20–50% of the resting energy expenditure of several freshwater fishes. The energetic cost to maintain osmatic balance is likely to increase in the future as the climate changes. Increased water temperature will result in a concomitant increase in metabolic rate for nearshore fishes, which increases food demands and foraging, which may alter predator–prey dynamics (Eme *et al.*, 2011; Kordas *et al.*, 2011). Finally, in extreme cases, prolonged exposure to sub-optimal water conditions can result in chronic stress for fishes, which can lead to reduced growth rates, reduced reproductive output and increased susceptibility to disease (Doney *et al.*, 2012). Together, results from the present study indicate that an acute change in temperature will be the most challenging component of future ocean conditions for nearshore fishes, particularly in the summer, with fish experiencing increased sub-lethal disturbances that could manifest in behavioural or habitat shifts.
